# Trial registration as a safeguard against outcome reporting bias and spin? A case study of randomized controlled trials of acupuncture

**DOI:** 10.1371/journal.pone.0223305

**Published:** 2019-10-03

**Authors:** Jiyoon Won, Seoyeon Kim, Inhu Bae, Hyangsook Lee

**Affiliations:** 1 Department of Korean Medical Science, Graduate School, Kyung Hee University, Dongdaemun-gu, Seoul, Republic of Korea; 2 Acupuncture & Meridian Science Research Centre, College of Korean Medicine, Kyung Hee University, Dongdaemun-gu, Seoul, Republic of Korea; 3 Kyung Hee University Korean Medicine Hospital, Dongdaemun-gu, Seoul, Republic of Korea; University of Zurich, SWITZERLAND

## Abstract

**Background and objective:**

Trial registration is widely endorsed as it is considered not only to enhance transparency and quality of reporting but also to help safeguard against outcome reporting bias and probably spin, known as specific reporting that could distort the interpretation of results thus mislead readers. We planned to investigate the current registration status of recently published randomized controlled trials (RCTs) of acupuncture, outcome reporting bias in the prospectively registered trials, and the association between trial registration and presence of spin and methodological factors in acupuncture RCTs.

**Methods:**

Acupuncture RCTs published in English in recent 5 years (January 2013 to December 2017) were searched in PubMed, Cochrane Central Register of Controlled Trials, and EMBASE. Trial registration records identified in the publications and trial registries were classified into prospectively registered, retrospectively registered, or unregistered. Primary outcomes were identified and the direction of the results was judged as statistically significant (positive) or statistically nonsignificant (negative). We compared registered and published primary outcomes to assess outcome reporting bias and assessed whether discrepancies favored statistically significant outcomes. Frequency and strategies of spin in published reports with statistically nonsignificant results for primary outcomes were then identified. We also analyzed whether the trial registration status was associated with spin and quality of methodological factors.

**Results:**

Of the 322 included RCTs, 41.9% (n = 135) were prospectively registered. Among 64 studies that were prospectively registered and specified primary outcomes, 25 trials had the discrepancies between the registered and published primary outcomes and 60% of them (15 trials) favored the statistically significant findings. Among 169 studies that specified primary outcomes, trial registration status was not associated with the direction of results, i.e., statistically significant or not. Spin was identified in 56.4% out of 78 studies with statistically nonsignificant primary outcomes and claiming efficacy with no consideration of statistically nonsignificant primary outcomes was the most common strategy for spin. Trial registration status was not statistically different between studies with and without spin.

**Conclusion:**

While trial registration seemed to have improved over time, primary outcomes in registered records and publications were often inconsistent, tending to favor statistically significant findings and spin was common in studies with statistically nonsignificant primary outcomes. Journal editors and researchers in this field should be alerted to still prevalent reporting bias and spin.

## I. Introduction

Research findings should be reported in complete, transparent, and accurate manners [1]. However, there are various obstacles to such reporting. Among them, outcome reporting bias in a clinical trial, defined as a selective reporting of outcomes influenced by the nature and direction of the results [1–4], has been widely recognized as one of the most substantial biases affecting the results from individual studies [5,6].

Separately from outcome reporting bias, a misrepresentation of study results regardless of intention, that overemphasizes the benefits of the intervention and exaggerates safety compared with those shown by the results, is known as spin [7–9]. Some researchers have recently demonstrated that when spin occurs it may mislead readers to inadequate interpretation and dissemination of research findings [10].

One of the recommended safeguards against outcome reporting bias and spin by securing the transparency of reporting is clinical trial registration, i.e., the systematic public disclosure of key descriptive information about a clinical trial before the commencement of study [11–13]. After the U.S. Food and Drug Administration (FDA) enacted legal mandates for registration of trials in 1997 [14], endorsement of trial registration before the onset of enrolling participants by the International Committee of Medical Journal Editors (ICMJE) followed [15,16]. Accordingly, the number of trial registration substantially increased [17,18] and in October 2018, ClinicalTrials.gov, the largest and most known trial registry, contains trial registration records of more than 280,000 studies from over 200 countries [19]. Despite requirements that trial registration should be completed before the first participant is enrolled at a publicly accessible trial registry, there have been concerns regarding inadequately registered studies [20]. Trials that were retrospectively registered or not registered have been reported more likely to overestimate the treatment effect than those prospectively registered [21]. Furthermore, regarding the quality of registered information, the discrepancies between information in the registry and publications such as changed, omitted, or newly introduced primary outcomes, have been identified [22,23] and such discrepancies are known to prevail across specialties [24–26].

Acupuncture is an important therapeutic modality in Traditional East-Asian Medicine and recently gaining popularity in Western countries [27,28]. As research on acupuncture rapidly increases over time [29], there is concerning voice that outcome reporting biases in acupuncture randomized controlled trials (RCTs) may hamper the reliability of the evidence. For example, though the number of registered acupuncture trials has increased, outcome reporting bias in acupuncture studies is still widespread and needs cautious interpretation similar to conventional medicine [30,31]. However, few studies have comprehensively examined the association between trial registration status and outcome reporting bias, still less with spin, i.e., representation of statistically nonsignificant outcomes with rhetoric shaping the opposite impression to readers [7,9,10].

In this context, this study planned to investigate the current registration status of acupuncture RCTs, to compare registered primary outcomes with published ones and to determine whether outcome reporting bias favors statistically significant results, and to investigate whether spin is prevalent and the trial registration is associated with spin.

## II. Methods

### 1. Search strategy

An electronic search in PubMed, EMBASE and Cochrane Controlled Register of Trials (CENTRAL) for RCTs of acupuncture published in the most recent 5 years from 2013 to 2017 was conducted. We developed a search strategy using key words regarding acupuncture modalities including manual acupuncture, electroacupuncture and ear acupuncture, and study design, i.e., RCT, with no language restriction. Full search strategies for each database can be found in S1 File.

### 2. Study selection

#### Study design

We defined an RCT as a prospective study in human participants who were randomly allocated to one of the study groups to assess beneficial, harmful, or physiologic effects of one or more healthcare interventions [2]. We only sought reports of parallel-group RCTs and accordingly, the other study designs such as case reports, case-control studies, observational studies, narrative or systematic reviews, editorials, commentaries or letters were excluded. Among RCTs, equivalence or non-inferiority trials, crossover trials, cluster trials, factorial and split-body design studies, trials with more than two groups, and phase 2 trials were not considered. In other words, we only included two group parallel RCTs to simplify evaluating process of statistical significance, i.e., p value less than 0.05, following the criteria of Boutron et al. [9], because determination of directionality and which arms to compare is usually difficult in > 2 arm studies and leaving out crossover studies for simplicity was considered appropriate [32,33]. We also excluded pilot and preliminary studies that inherently do not specify primary outcomes and brain imaging studies using functional magnetic resonance imaging, computed tomography, and positron emission tomography that usually do not specify primary outcomes and investigate an intervention group’s statistical significance over a control group. However, we considered RCTs using brain imaging in addition to an adequate clinical outcome measure such as visual analogue scale (VAS) or blood pressure.

#### Intervention

We included both invasive acupuncture needling and non-invasive interventions applied to acupoint(s), i.e., acupressure, moxibustion and laser acupuncture, that are defined as acupuncture in the World Health Organization (WHO) [34]. When acupuncture treatment was tested in combination with other treatment(s), only studies where the identical co-intervention was applied to both groups were considered. In the comparator group were encompassed active controls such as standard treatment and inactive controls such as no treatment, waiting list or sham control.

#### Selection procedure

Two authors (S. Kim and I. Bae) searched the electronic databases, screened the eligibility of studies based on the title, abstract, and existence of full text publications. Conference abstract and trial registration records of ClinicalTrials.gov. were excluded accordingly. Included full text articles in English were independently read in full and assessed for eligibility by the two authors (J. Won and S. Kim). Discrepancies were resolved by discussion with the corresponding author (H. Lee).

### 3. Trial registration status

We manually screened the trial registration number in the main text of the publication. If the registration number was not specified in the publication, we searched the WHO trial registry portal [35] using the details of authors’ name, subject of the trial, trial title or source of funding [36]. We classified the trial registration status into 1) prospectively registered; 2) retrospectively registered according to the registration date; or 3) unregistered. Prospective registration was defined as trial registration before or within a month of the 1st participant enrollment start date according to the protocol [21], in consideration of guidelines on trial registration from ICMJE that states trial registration should be done before enrolling patients [37] and FDA where trial registration is required within 21 days of enrollment of the 1st participant [38]. Thus, retrospective registration was defined as trial registration over one month after the participant enrollment, i.e., start date.

### 4. Data extraction

The full texts of a random sample of 10 published articles were read by three authors (S. Kim, I. Bae and J. Won) independently and the following information was extracted and tabulated in the Microsoft Excel sheet (Microsoft Corporation, Redmond, WA, USA): journal name/type, study origin, sample size, primary outcome and direction of the results based on the reported primary outcome (statistically significant or not). If there was any disagreement during the initial data extraction among three authors, it was resolved by discussion with the corresponding author (H. Lee). Then, data were extracted from the remaining 312 articles following the identical procedure described above.

We dichotomized journal type into complementary and alternative medicine (CAM) journals and non-CAM journals based on the subject category in Scopus^®^. We also examined whether the journals where the included studies were published endorsed trial registration by specifying mandatory trial registration in author guidelines or not. The study origin was defined as the country where the study was conducted or the country where the affiliation of the first author belonged to if the study was a multi-national one and the extracted data on study origin was then aggregated into East Asia or non-East Asia. This was based on the previous report that showed studies from East Asian countries were more likely to report statistically significant results than those from non-East Asian countries in the field of acupuncture research [39]. We presented sample size of each trial with median and interquartile range and publication year of studies over from 2013 to 2017. Control groups of RCTs were divided into 4 types: sham acupuncture; active treatment such as drug, behavioral therapy, physical therapy, standard care or other relevant treatment; no treatment; or other type of acupuncture. Primary outcomes were extracted only when they were explicitly specified in the publication. If none was clearly specified, we regarded an outcome used in calculating sample size as a primary outcome [23]. When we identified neither explicitly reported primary outcomes nor the outcome used in the sample size calculation, we considered the article did not specify primary outcomes. The direction of the result was dichotomized as statistically significant or statistically nonsignificant. The direction of result was considered statistically nonsignificant if the result favors control against the intervention or at least one primary outcome was not statistically significant when there were more than one primary outcomes.

We used the Cochrane Collaboration’s risk of bias assessment tool to evaluate likelihood of selection bias (random sequence generation and allocation concealment), performance bias (participant blinding), detection bias (outcome assessment blinding), attrition bias (incomplete outcome data), and reporting bias (selective outcome reporting) [6]. Given that practitioner blinding is virtually impossible in acupuncture trials, blinding of only participants and outcome assessment was evaluated. Risk of bias was determined as having a low or high/unclear risk for each item for individual studies. Selective outcome reporting was rated low when the study registration record was available and all of the study’s pre-specified outcomes were reported in the pre-defined way.

Details of trial registration status including the name of trial registry, type of trial registration, i.e., prospective, retrospective, or no registration, and registered primary outcomes were also extracted. Regarding the registered primary outcome, if the detailed information in the registry was modified after the start of the study, we extracted only the initially registered primary outcome.

### 5. Data analyses

#### Outcome reporting bias

To examine the presence of outcome reporting bias, we compared the specified primary outcomes in the trial registry with those reported in the publication. We identified alteration of the primary outcomes only for the prospectively registered studies since we deemed it inappropriate to evaluate primary outcomes’ modification in retrospectively registered studies [23]. If the primary outcomes in the trial registry differed from those in the published articles, we categorized the types of discrepancy according to Mathieu et al. as follows [23]: 1) primary outcomes in a registry were described as secondary in the publications; 2) primary outcomes in a registry were omitted in a publication; 3) primary outcomes in a publication were originally secondary outcomes in a registry; 4) new primary outcomes were adopted in a published article; and/or 5) the timing of assessment in a publication differed from the timing of assessment in a registry. It was also assessed whether the discrepancy was favorable to the statistically significant results. We judged the discrepancy was ‘favoring statistically significant results’ when 1) the primary outcomes in a registry were not reported as such in a published article and they were not statistically significant; and/or 2) the outcomes altered from secondary to primary and/or newly adopted ones as primary in the publication had statistically significant results. When it was impossible to assess the direction of results of the registered primary outcomes because they were omitted in a publication, it was categorized into ‘impossible to conclude’.

#### Spin

Spin is defined as a specific way of reporting, intentionally or not, to describe the results as effective or beneficial even though they were not positive [9,10,40,41]. The presence of spin was identified only in studies having statistically nonsignificant primary outcomes. When a study reported that an intervention was effective or beneficial in conclusion section of the abstract despite the statistically nonsignificant primary outcome, it was considered as involving spin [40]. The types of spin were determined according to Boutron et al.’s classification as follows [9]: claiming equivalence or comparable effectiveness for statistically nonsignificant results; claiming efficacy with no consideration of statistically nonsignificant primary outcomes; focusing only on statistically significant results; acknowledging statistically nonsignificant results for the primary outcomes but emphasizing the beneficial effect of treatment; acknowledging statistically nonsignificant results for the primary outcome but emphasizing other statistically significant results; conclusion focusing on within-group comparisons; recommendation to use the treatment; focusing on another objective; comparing with placebo group of another trial; and statistically nonsignificant subgroup results reported as beneficial. As the type of control group was considered to be associated with categorization of spin [9], we searched the comparator in the publication with statistically nonsignificant primary outcomes.

#### Association between trial registration status and study results and methodological factors

In the present study, the association between the trial registration status and the direction of primary outcomes, i.e., statistically significant or not, was investigated only in the studies that specified the primary outcomes meeting the aforementioned criteria, since the judgement of the direction of study result was considered valid only when the primary outcome was explicitly defined and reported. Trial registration status was dichotomized under two scenarios: 1) ‘registered’ (both prospectively and retrospectively registered) vs. ‘unregistered’; and 2) ‘prospectively registered’ vs. ‘unregistered or retrospectively registered'.

Whether trial registration status was different between CAM and non-CAM journals (journal specialty), between mandatory trial registration in the journal’s author guidelines and optional registration (mandatory trial registration), and between study origins was explored. Median sample size was also compared between different trial registration scenarios. The association between the two trial registration scenarios and quality of methodological factors such as random sequence generation, allocation concealment, blinding of participants, blinding of outcome assessment, incomplete outcome data, and selective outcome reporting was examined.

#### Statistical analysis

All statistical analyses involved the use of Statistical Analysis System (SAS) package (version 9.4. SAS Institute Inc., Cary, NC, USA). Change in the proportion of registered vs. unregistered studies and prospectively registered vs. retrospectively registered or unregistered studies during publication year from 2013 to 2017 was evaluated using Cochran-Armitage trend test. The association between trial registration status and proportion of the studies with spin vs. no spin, statistically significant vs. statistically nonsignificant results, journal specialty, mandatory trial registration, study origin, and risk of bias was analyzed by Fisher’s exact test. In addition, median sample size was compared with Mann-Whitney U test between registration status, i.e., registered vs. unregistered, and prospectively registered vs. unregistered and retrospectively registered [24]. All statistical analyses were considered significant when P value was less than 0.05.

## III. Results

### 1. Search results

**Fig 1:**
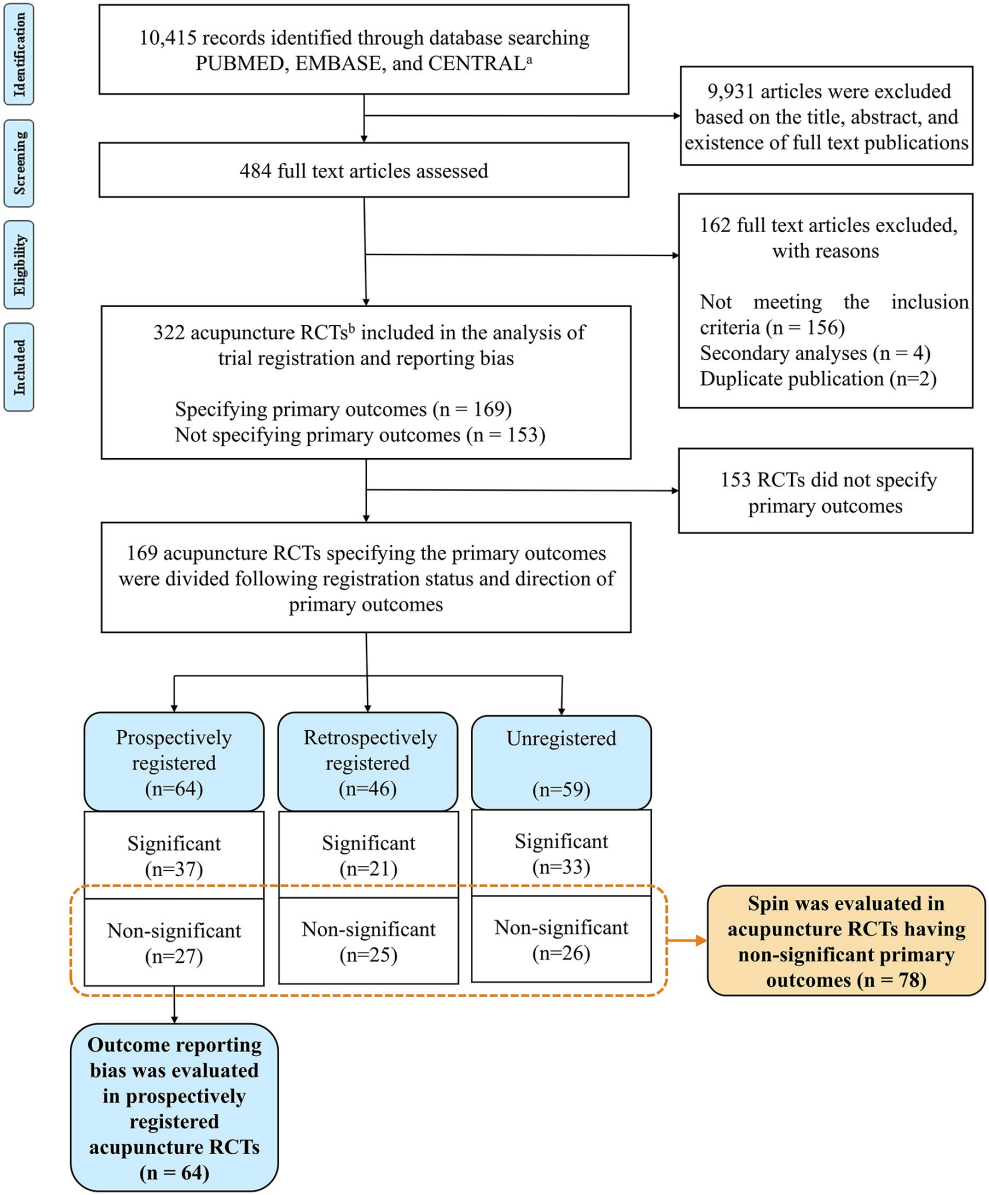
Flow diagram of study selection. (a) Cochrane Controlled Register of Trials (b) Randomized controlled trials.

A total of 10,415 articles were retrieved through electronic database search. After excluding 9,931 articles based on the title and abstract, 484 articles were included in the full text review. Of these, 322 articles met the inclusion criteria and were finally included in the analysis (Fig 1).

### 2. Characteristics and risk of bias of the included studies

Characteristics and risks of bias of the included studies are presented in Table 1. Of the 322 included articles, less than half of them (135 studies, 41.9%) were registered and for the other 187 studies (58.1%), registration numbers were neither identified in the publication nor WHO trial registry portal: of the 135 registered trials, 74 studies (54.8%) were prospectively registered, and 61 (45.2%) retrospectively registered; 117 reported the registration records, and 18 were identified in the WHO trial registry. Among 117 trial registration numbers in the published studies, two of them were recorded incorrectly. Studies were registered in 10 different registries and two registries had more than half of the registrations (89 studies, 65.5%): 61 (44.9%) in the ClinicalTrials.gov registry, and 28 (20.6%) in the Chinese Clinical Trial Registry (ChiCTR) (S1 Table).

**Table 1:** Characteristics and risk of bias of the included acupuncture RCTs (n = 322).

Number (%) of studies					
	Registered			Unregistered	Total
	Prospectively registered	Retrospectively registered	Registered total		
	(n = 74)	(n = 61)	(n = 135)	(n = 187)	(n = 322)
Journal specialty					
CAM Journal	25 (33.8)	30 (49.2)	55 (40.7)	115 (61.5)	170 (52.8)
Non-CAM Journal	49 (66.2)	31 (50.8)	80 (59.3)	72 (38.5)	152 (47.2)
Mandatory trial registration					
Mandatory	48 (64.9)	30 (49.2)	78 (57.8)	93 (49.7)	171 (53.1)
Not mandatory	26 (35.1)	31 (50.8)	57 (42.2)	94 (50.3)	151 (46.9)
Study origin					
East Asia	36 (48.6)	24 (39.3)	60 (44.4)	119 (63.6)	179 (55.6)
Non-East Asia	38 (51.4)	37 (60.7)	75 (55.6)	68 (36.4)	143 (44.4)
Sample size					
Median (IQR)	80.5 (55.5–128)	80 (47–130)	80 (53–129)	60 (42.5–80.5)	63 (48.5–100)
Year of publication^a^					
2013	13 (17.6)	13 (21.3)	26 (19.2)	40 (21.4)	66 (20.5)
2014	5 (6.7)	12 (19.7)	17 (12.6)	42 (22.5)	59 (18.3)
2015	13 (17.6)	11 (18.0)	24 (17.8)	50 (26.7)	74 (23.0)
2016	20 (27.0)	14 (23.0)	34 (25.2)	32 (17.1)	66 (20.5)
2017	23 (31.1)	11 (18.0)	34 (25.2)	23 (12.3)	57 (17.7)
Types of control group					
Sham acupuncture	35 (47.3)	32 (52.5)	67 (49.6)	63 (33.7)	130 (40.4)
Active treatment^b^	21 (28.4)	25 (41.0)	46 (34.1)	76 (40.6)	122 (37.9)
No treatment	11 (14.9)	3 (4.9)	14 (10.4)	17 (9.1)	31 (9.6)
Acupuncture	7 (9.4)	1 (1.6)	8 (5.9)	31 (16.6)	39 (12.1)
Reported primary outcomes					
Specified	64 (86.5)	46 (75.4)	110 (81.5)	59 (31.6)	169 (52.5)
Not specified	10 (13.5)	15 (24.6)	25 (18.5)	128 (68.4)	153 (47.5)
Random sequence generation^c^					
Low risk	62 (83.8)	43 (70.5)	105 (77.8)	113 (60.4)	218 (67.7)
High or unclear risk	12 (16.2)	18 (29.5)	30 (22.2)	74 (39.6)	104 (32.3)
Allocation concealment^c^					
Low risk	47 (63.5)	27 (44.3)	74 (54.8)	38 (20.3)	112 (34.8)
High or unclear risk	27 (36.5)	34 (55.7)	61 (45.2)	149 (79.7)	210 (65.2)
Blinding of participants^c^					
Low risk	36 (48.6)	34 (55.7)	70 (51.9)	64 (34.2)	134 (41.6)
High or unclear risk	38 (51.4)	27 (44.3)	65 (48.1)	123 (65.8)	188 (58.4)
Blinding of outcome assessment^c^					
Low risk	45 (60.8)	35 (57.4)	80 (59.3)	57 (30.5)	137 (42.5)
High or unclear risk	29 (39.2)	26 (42.6)	55 (40.7)	130 (69.5)	185 (57.5)
Incomplete outcome data^c^					
Low risk	68 (91.9)	54 (88.5)	122 (90.4)	148 (71.5)	270 (83.9)
High or unclear risk	6 (8.1)	7 (11.5)	13 (9.6)	39 (28.5)	52 (16.1)
Selective outcome reporting^c^					
Low risk	39 (52.7)	-	39 (28.9)	-	39 (12.1)
High or unclear risk	35 (47.3)	61 (100.0)	96 (71.1)	187 (100.0)	283 (87.9)

RCTs, randomized controlled trials; CAM, complementary and alternative medicine; IQR, Interquartile range.

^a^The proportion of registered studies and prospectively registered studies showed a significant increasing trend over publication year from 2013 to 2017 (the Cochran-Armitage trend test, P<0.05 and P<0.001, respectively).

^b^In active treatment was included drug, behavioral therapy, physical therapy, standard care and other relevant treatment.

^c^All risk of bias assessment was done using the Cochrane risk of bias assessment tool [6].

The proportion of registered trials published in CAM journals (55 of 170 studies, 32.4%) was lower than that of non-CAM journals (80 of 152 studies, 52.6%; P = 0.0002). Whether trial registration was mandatory or not in the author guidelines did not seem to be associated with the registration status of the included studies (P = 0.15). Of 179 studies conducted in East Asia, only 60 studies (33.5%) were confirmed to have been registered; of 143 studies conducted in non-East Asia, 75 studies (52.4%) were registered. Median sample size of the included studies was 63 (interquartile range 48.5–100): registered studies had the median sample size of 80.5 (55.5–128) and unregistered studies had 80 (47–130). Over the past 5 years, the proportion of registered trials has increased and that of prospectively registered trials also has increased significantly (Cochran-Armitage trend test, P<0.05 and P<0.001 respectively). Regardless of registration status, most of included studies (252 studies, 78.3%) used sham acupuncture and active treatment having a physiologic effect as comparators. While 110 out of 135 (80.9%) registered studies specified the primary outcomes in their publications, only 31.6% (59 out of 187) of unregistered trials did so.

Regarding risk of bias, the proportion of prospectively registered studies with a low risk of bias in all 6 domains was 16.2% (12 of 74). All retrospectively registered and unregistered studies were given unclear or high risk of bias for selective outcome reporting. Results of risk of bias assessment for all the included studies are described in S2 Table.

### 3. Outcome reporting bias

A total of 64 studies that had been prospectively registered and specified primary outcomes were included in this analysis [23]. The discrepancy between registered and published primary outcomes was identified in 25 out of 64 studies (39.1%). Types of discrepancies varied (Table 2) and 8 reports involved two different types of discrepancies and one article had three types of discrepancies thus 35 discrepancies were identified. The most common discrepancy was that the registered primary outcomes were reported as secondary ones in the publication (16 out of 35 reports, 45.7%): a study assessing the additional effects of acupuncture on early rehabilitation in patients with acute ischemic stroke registered mortality, National Institutes of Health Stroke Scale (NIHSS), Fugl-Meyer Assessment (FMA), Mini-Mental State Examination (MMSE), Montreal Cognitive Assessment (MoCA), depression cropland of swallowing ability evaluation as primary outcomes whereas only NIHSS was described as a primary outcome and other registered primary outcomes were reported as secondary outcomes in a publication.

**Table 2:** Concordance of registered and published primary outcomes in acupuncture RCTs prospectively registered and specified primary outcomes (n = 64).

	Number (%) of articles
	(n = 64)
Concordant	39 (60.9)
Discordant	25 (39.1)
Types of discrepancies	25^a^
Primary outcomes in a registry were described as secondary in the publications	16 (25.0)
Primary outcomes in a registry were omitted in a publication	6 (9.4)
Primary outcomes in a publication were originally secondary outcomes in a registry	5 (7.8)
New primary outcomes were adopted in a published article	4 (6.3)
The timing of assessment in a publication differed from the timing of assessment in a registry	4 (6.3)
Discrepancies in primary outcomes favoring statistically significant results	25
Yes	15 (60.0)
No	6 (24.0)
Impossible to conclude^b^	4 (12.0)

RCTs, randomized controlled trials.

^a^Eight articles involved two types of discrepancies and one article involved three types of discrepancies.

^b^Four studies included registered primary outcomes that were omitted in the published articles.

Of the 25 studies with the primary outcome discrepancy, 15 studies (60.0%) were judged that the discrepancy favored statistically significant results, possibly implying outcome reporting bias. For example, in a study aiming to evaluate clinical efficacy and safety of acupuncture treatment for primary insomnia, insomnia severity index (ISI) and sleep parameters such as sleep efficiency, sleep awakenings, and total sleep time were registered as primary outcomes. In the published article, however, sleep parameters with no statistical significance were reported as secondary outcomes.

### 4. Spin

Out of 169 studies that specified the primary outcomes, 78 studies with statistically nonsignificant results were reviewed for the identification of spin (Table 3). The presence of spin in the conclusion section of abstract was detected in 44 out of 78 studies (56.4%). Out of 44 studies with spin, the most prevalent spin type was to claim efficacy with no consideration of statistically nonsignificant primary outcomes, identified in 23 studies (52.3%). For example, a study investigating the difference of clinical pain intensity as a primary outcome between verum and sham acupuncture in fibromyalgia concluded the efficacy of acupuncture seemed underestimated even though the outcome did not significantly differ from the control group.

**Table 3:** Presence and types of identified spin in acupuncture RCTs with statistically nonsignificant primary outcomes (n = 78).

	Number (%) of articles (n = 78)				
	Prospectively registered	Retrospectively registered	Registered total	Unregistered	Total
	(n = 27)	(n = 25)	(n = 52)	(n = 26)	(n = 78)
No spin	14 (51.9)	11 (44.0)	25 (48.1)	9 (34.6)	34 (43.6)
Spin	13 (48.1)	14 (56.0)	27 (51.9)	17 (65.4)	44 (56.4)
Types of spin^a^					
Focusing only on treatment effectiveness	7 (25.9)	11 (44.0)	18 (34.6)	17 (65.4)	35 (44.9)
Claiming equivalence or comparable effectiveness for statistically nonsignificant results	3 (11.1)	1 (4.0)	4 (7.7)	2 (7.7)	6 (7.7)
Claiming efficacy with no consideration of statistically nonsignificant primary outcomes	4 (14.8)	8 (32.0)	12 (23.1)	11 (42.3)	23 (29.5)
Focusing only on statistically significant results	-	2 (8.0)	2 (3.8)	4 (15.4)	6 (7.7)
Acknowledging statistically nonsignificant results for the primary outcomes but emphasizing the beneficial effects of treatment	3 (11.1)	-	3 (5.8)	-	3 (3.8)
Acknowledging statistically nonsignificant results for the primary outcomes but emphasizing other statistically significant results	3 (11.1)	1 (4.0)	4 (7.7)	-	4 (5.1)
Recommendation to use the treatment	-	2 (8.0)	2 (3.8)	-	2 (2.6)

RCTs, randomized controlled trials.

^a^Boutron et al.’s classification [9] was adopted.

The overall proportion of registered studies with spin (27 of 52 studies, 52.0%) was slightly lower than that of unregistered studies with spin (17 of 26 studies, 65.4%) but there was no significant difference (Fisher’s exact test, P = 0.335). Among the identified 6 different types of spin, three types of spin, i.e., ‘acknowledging statistically nonsignificant results for the primary outcomes but emphasizing the beneficial effects of treatment', ‘recommendation to use the treatment', and ‘acknowledging statistically nonsignificant results for the primary outcomes but emphasizing other statistically significant results’ were detected only in registered studies: one study evaluating the effect of acupuncture for ovulation induction in polycystic ovary syndrome had a statistically nonsignificant primary outcome (LH pulsatility) but highlighted higher ovulation frequency in the acupuncture group than in the control.

### 5. Association between trial registration status and study results and methodological factors

Studies specifying primary outcomes (n = 169) were all identified as evaluating efficacy of acupuncture. Among them, the proportion of studies reporting statistically significant results did not statistically differ regardless of trial registration, i.e., registered vs. unregistered and prospectively registered vs. unregistered or retrospectively registered (Table 4).

**Table 4:** Association between trial registration status and study results and methodological factors in acupuncture RCTs specifying primary outcomes (n = 169).

	Registered vs. unregistered			Prospectively registered vs. unregistered or retrospectively registered		
	Number (%) of articles			Number (%) of articles		
	Registered^a^	Unregistered	*P value*	Prospectively registered	Unregistered or retrospectively registered	*P value*
	(n = 110)	(n = 59)		(n = 64)	(n = 105)	
Direction of primary outcomes^b^						
Statistically significant	58 (52.7)	33 (55.9)	0.747	37 (57.8)	54 (51.4)	0.432
Statistically nonsignificant	52 (47.3)	26 (44.1)		27 (42.2)	51 (48.6)	
Journal specialty^b^						
CAM Journal	39 (35.5)	32 (54.2)	0.022	19 (29.7)	52 (49.5)	0.016
Non-CAM Journal	71 (64.5)	27 (45.8)		45 (70.3)	53 (50.5)	
Mandatory trial registration^b^						
Mandatory	67 (60.9)	26 (44.1)	0.051	44 (68.8)	49 (46.7)	0.007
Not mandatory	43 (39.1)	33 (55.9)		20 (31.2)	56 (53.3)	
Study origin^b^						
East Asia	50 (45.5)	30 (50.8)	0.522	31 (48.4)	49 (46.7)	0.874
Non-East Asia	60 (54.5)	29 (49.2)		33 (51.6)	56 (53.3)	
Sample size^c^						
Median (IQR)	82 (54.5–153.5)	62 (50–97.5)	0.011	81.5 (54.5–151)	72 (50–120)	0.083
Random sequence generation^b^						
Low risk	93 (84.6)	40 (67.8)	0.017	57 (89.1)	76 (72.4)	0.012
High or unclear risk	17 (15.4)	19 (32.2)		7 (10.9)	29 (27.6)	
Allocation concealment^b^						
Low risk	70 (63.6)	21 (35.6)	0.001	46 (71.9)	45 (42.9)	<0.001
High or unclear risk	40 (36.4)	38 (64.4)		18 (28.1)	60 (57.1)	
Blinding of participants^b^						
Low risk	60 (54.5)	31 (52.5)	0.872	32 (50.0)	59 (56.2)	0.525
High or unclear risk	50 (45.5)	28 (47.5)		32 (50.0)	46 (43.8)	
Blinding of outcome assessment^b^						
Low risk	71 (64.6)	31 (52.5)	0.140	42 (65.6)	60 (57.1)	0.331
High or unclear risk	39 (35.4)	28 (47.5)		22 (34.4)	45 (42.9)	
Incomplete outcome data^b^						
Low risk	102 (92.7)	50 (84.7)	0.113	60 (93.8)	92 (87.6)	0.292
High or unclear risk	8 (7.3)	9 (15.3)		4 (6.2)	13 (12.4)	
Selective outcome reporting^b^						
Low risk	39 (35.5)	-	<0.001	39 (60.9)	-	<0.001
High or unclear risk	71 (64.5)	59 (100.0)		25 (39.1)	104 (100.0)	

RCTs, randomized controlled trials; CAM, complementary and alternative medicine; IQR, interquartile range.

^a^Registered included both prospectively and retrospectively registered studies.

^b^Association between registration status and methodological factors was analyzed using Fisher’s exact test.

^c^Association between registration status and sample size was subject to Mann-Whitney U test.

Compared to studies in non-CAM journals, studies published in CAM journals were more likely to be retrospectively registered or unregistered (P<0.05); studies published in journals that do not endorse mandatory trial registration were also more likely to be registered retrospectively or not to be registered in the public registry than those published in journals that mandate trial registration (P = 0.007), but such association was no longer significant when analyzed in the classification of registered vs. unregistered (P = 0.051). Trial registration status did not differ between regions where the study was conducted, i.e., East-Asian and non-East Asian countries. Median value of sample size was not significantly different between prospective registered and retrospectively or unregistered studies whereas it turned out to be significantly larger in registered studies than unregistered studies (82 vs. 62, P<0.05).

Random sequence generation, allocation concealment, and selective outcome reporting were more likely to be adequately done in registered studies than in unregistered ones (P<0.05). Compared with retrospectively or unregistered studies, prospectively registered studies were also more likely to have a low risk of bias in the above three domains (P<0.05). In contrast, no such association appeared in blinding of participants, blinding of outcome assessments and incomplete outcome reporting.

## IV. Discussion

### 1. Summary of main findings

In this study, we looked at the current registration status of acupuncture RCTs; compared prospectively registered primary outcomes with published ones to determine whether outcome reporting bias favors statistically significant results; and the trial registration status was associated with spin, i.e. distorted interpretation of statistically nonsignificant results.

Whereas trial registration rate has significantly improved over the recent 5 years, of 322 acupuncture RCTs included in our study, only 41.9% registered their studies and even less studies (22.3%) did so prospectively (registered before or within one month after the 1st participant enrollment start date). Outcome reporting bias or the discrepancy of primary outcomes between the trial registration record and publication, was identified in 39.1% of the prospectively registered studies which specified primary outcomes, the most common discrepancy being outcome changed from primary to secondary. Similar to previous findings that studies with discrepancies between registered primary outcomes and those published favored statistically significant outcomes [2,36,42], such association was also found in our study. Spin was identified in more than half of the studies with statistically nonsignificant outcomes (56.4%). Among various strategies of spin, claiming efficacy with no consideration of statistically nonsignificant primary outcomes was the most common.

Retrospectively registered or unregistered studies were found more likely to be published in CAM journals and also journals that do not endorse mandatory trial registration in their instructions for authors. Trial registration status was not different between studies conducted in East Asia and the other regions or studies where primary outcomes favored statistically significant results and those that did not. Compared to that of retrospectively or unregistered studies, random sequence generation, allocation concealment, and selective outcome reporting was considered more adequately done in (prospectively) registered trials whereas blinding of participants, blinding of outcome assessment, and incomplete outcome data did not differ by registration status.

### 2. Interpretation and implication of study findings

Trial registration is deemed a useful tool for authors and readers to build confidence in research findings by reporting outcomes transparently and accurately before the commencement of the trial [15]. We identified a significantly increasing trend not only for trial registration but also for the prospective registration of acupuncture RCTs and this is in line with the proportion of registered studies also on the rise across medical specialties [18,43]. Nevertheless, still a relatively low proportion of prospective registration (23.2%) and omission or incorrect reporting of trial registration number in the final publication, i.e., simple typing errors of registration numbers or incorrect spacing between the name of the registry and number that makes searching difficult, leave much to be desired. Furthermore, retrospective registration or missing registration number in the subsequent publication is not uncommon [23,36,42], e.g., authors omit registration numbers in the publications against the CONSORT statement while they report it in the protocol articles [1,44], and such inadequate practices hinder the original purposes of trial registration [37]. Timing and reporting of trial registration still needs improvement in acupuncture trials just like in clinical trials in general.

In addition to publication bias, outcome reporting bias has widespread impact as it increases the prevalence of spurious findings, and leads to misleading conclusions when selectively reported statistically significant outcomes are aggregated in systematic reviews and meta-analyses [2,45]. Previous studies have reported on outcome reporting biases in various medical subspecialties and outcome discrepancies between study protocols and published articles were not ignorable, as low as 5% to as high as 71% [2,25,30,36,42]. Similar to these findings, our study also found approximately 40% of prospectively registered studies with specified primary outcomes had discrepancies between registered and reported outcomes. More importantly, considering that a previous study reported statistically significant outcomes had higher odds of being fully reported than statistically nonsignificant outcomes [2] and many other reports including ours equally confirm outcome reporting bias favors statistically significant findings, a more careful scrutiny of trial registration by peer reviewers and journal editors is needed as the last gatekeepers to outcome reporting bias/selective outcome reporting to favor study intervention [36,42].

We found that in more than half (56.4%) of the included acupuncture RCTs with statistically nonsignificant primary outcomes, had a spin in their reporting in the abstract conclusion. The dominant type was to focus only on treatment effectiveness, which was consistent with the previous findings [9]. While Boutron et al. reported that the most common subcategory of spin was to claim equivalence or comparable effectiveness for statistically nonsignificant outcomes [9], ‘claiming efficacy with no consideration of statistically nonsignificant primary outcomes’ was the most common in our analysis. This may be partly because approximately 44.4% of the included trials had an active comparator group in Boutron et al.’s report [9], compared to 21.8% in our analysis. Although trial registration has been suggested as one of countermeasures against spin [12], we argue that it might not be a perfect solution to spin as the proportion of studies with spin was not associated with trial registration status.

Our analysis showed that direction of primary outcomes, i.e., statistically significant or not, doesn’t seem to be related with the registration status. Previous studies evaluating association between trial registration and treatment effect size have reported inconsistent results [21,46] and another recent study reported that direction of primary outcome was not subject to the status of registration [47]. Given these inconsistent results, a larger meta-epidemiological and/or cross-sectional study encompassing various medical disciplines is needed to determine the association between trial registration and treatment effect size and/or direction of study outcomes. Nevertheless, in light of our finding that studies published in journals that do not endorse mandatory trial registration were more likely to be unregistered or retrospectively registered, trial registration endorsement or specification in author guidelines by individual journals are still warranted as a safeguard to outcome reporting bias.

### 3. Strengths and limitations of this study

To the best of our knowledge, this is the first study that investigated the association between trial registration status and prevalence of spin in published acupuncture RCTs. Nevertheless, we acknowledge that this study is subject to several limitations. First, we only included articles published in English. Some might argue that inclusion of Chinese language articles may have introduced a different picture. While approximately 2,000 acupuncture RCTs have been published in Chinese journals over the past 20 years [48], a recently reported trial registration rate of articles published in Chinese journals (1.4%) [49], leaves much to be desired compared to that of our study (41.9%). Our study mainly focused on outcome reporting bias in the prospectively registered studies and the association between trial registration status and spin. Thus, inclusion of few registered Chinese language publications could unnecessarily lower trial registration rate in our study, and also may fail to represent current Chinse acupuncture RCTs. In addition, inclusion of too many unregistered Chinese language publications may result in imbalanced comparison in our analysis regarding spin. Nevertheless, it would be an interesting topic if acupuncture RCT reports published in Chinese journals are put under scrutiny for the association between trial registration and outcome reporting bias/spin when trial registration gets more generalized in China. Then as a next step, we can answer whether inclusion of Chinese publications would yield a different picture.

Second, although we extensively searched publication records and WHO trial registry portal to obtain registration number, we may not be completely free from missing some registered trials or incorrectly categorizing some registered trials as unregistered. Given that not all journal editors and publishers enforce or are advocates for trial registration [50] and a recent survey of RCTs published in Medline in 2010 found that 61% were registered but only 55% of the published articles specified trial registration numbers [44], our limitation may have been hard to avoid completely. Third, identification and classification of spin necessarily involves subjective judgement and therefore we cannot be free from interpretation bias [51] in assessing spin. To overcome this limitation, we tried to minimize subjectivity by reaching consensus on the category of various spin strategies among three authors and if there was any disagreement, it was resolved by discussion with the corresponding author. Also we adopted Boutron et al.’s classification of spin strategies [9] which is regarded as the most widely accepted tool so far [52].

### 4. Implication for research and practice

Our study did not find that unregistered trials were more likely to spin their results than registered trials. Given that outcome reporting bias and spin are still prevalent [24,25,40] even in medical specialties with relatively high proportion of trial registration such as critical care [53], oncology [25], and cardiology [24] field, trial registration alone may not be so much a panacea as expected [12]. Nonetheless, trial registration is essential for comparison between protocol and published article to detect outcome reporting bias and spin in the published reports. After adequate registration with details of research objectives, inclusion/exclusion criteria, primary and secondary outcomes, sample size calculation, analysis plan, and methodological factors related with risk of bias, authors should be cautious not to misinterpret the data [51], and provide the publicly accessible trial registration number in the published article for readers in the correct manner. The academic organizations where authors are affiliated to should provide relevant policies and resources to register trials or may even penalize researchers for non-compliance [54]. Journal editors and peer reviewers subject to concern of not detecting spin completely [55] can be systematically trained and provided with proper checklists or tools [56].

## V. Conclusion

The proportion of trial registration of acupuncture RCTs significantly improved, while it is not to a desirable level. About 40% of prospectively registered studies showed discordance between the registered and published primary outcomes and 60% of those with discrepancy favored the results. More than half of trials with statistically nonsignificant primary outcomes tended to interpret the results as effective with no consideration of statistical significance. Though trial registration is regarded as a powerful safeguard against inaccurate reporting, this study implies registration alone cannot be an answer. More attention from journal editors and researchers in this field is warranted.

## Supporting information

S1 Table Information of Registries where the included acupuncture RCTs^a^ were registered. ^a^Randomized controlled trials; ^b^One article was registered in two registries, ANZCTR and ReBec.
(DOCX)

S2 Table Risk of bias assessment of included studies. (XLSX)

S1 File Full search strategies for PubMed, EMBASE, and Cochrane CENTRAL. (DOCX)

S2 File Data of included acupuncture randomized controlled trials. (XLSX)

S3 File PRISMA checklist. (DOCX)
