# Supplementary material for: Trial registration as a safeguard against outcome reporting bias and spin? A case study of randomized controlled trials of acupuncture
Source: PLoS One. 2019 Oct 3;14(10):e0223305. doi: 10.1371/journal.pone.0223305 (PMC6776391; doi:10.1371/journal.pone.0223305)
Supplement: S1 Table — aRandomized controlled trials; bOne article was registered in two registries, ANZCTR and ReBec. (DOCX) [file pone.0223305.s001.docx]

**[S1 Table]** Information of Registries where the included acupuncture RCTs were registered

|  | Number (%) of articles | | |
| --- | --- | --- | --- |
|  | Prospectively registered^a^ | Retrospectively registered | Total^a^ |
|  | (n = 75) | (n = 61) | (n = 136) |
| ClinicalTrials.gov | 32 (42.7) | 29 (47.6) | 61 (44.9) |
| Chinese Clinical Trials Registry (ChiCTR) | 21 (28.0) | 7 (11.5) | 28 (20.6) |
| Iranian Registry of Clinical Trials (IRCT) | 6 (8.0) | 10 (16.4) | 16 (11.8) |
| Australian New Zealand Clinical Trials Registry (ANZCTR) | 9 (12.0) | 1 (1.6) | 10 (7.3) |
| International Standard Randomised Controlled Trial Number Registry (ISRCTN) | 1 (1.3) | 7 (11.5) | 8 (5.9) |
| Brazilian Clinical Trials Registry (Registro Brasileiro de Ensaios Clinicos, ReBec) | 2 (2.7) | 3 (4.9) | 5 (3.7) |
| German Clinical Trials Register (Das Deutsche Register Klinischer Studien, DRKS) | 1 (1.3) | 2 (3.3) | 3 (2.2) |
| Clinical Research Information Service, Republic of Korea (CRIS) | 2 (2.7) | 1 (1.6) | 3 (2.2) |
| Thai Clinical Trials Registry (TCTR) | 1 (1.3) | - | 1 (0.7) |
| European Clinical Trials Database (EudraCT) | - | 1 (1.6) | 1 (0.7) |

RCTs, randomized controlled trials.

^a^One article was prospectively registered in two registries, ANZCTR and ReBec.
