# Supplementary material for: Trial registration as a safeguard against outcome reporting bias and spin? A case study of randomized controlled trials of acupuncture
Source: PLoS One. 2019 Oct 3;14(10):e0223305. doi: 10.1371/journal.pone.0223305 (PMC6776391; doi:10.1371/journal.pone.0223305)
Supplement: S1 File — (DOCX) [file pone.0223305.s003.docx]

**[S1 File]** Full search strategies for PubMed, EMBASE, and Cochrane CENTRAL.

1. **PubMed**

(("acupuncture"[MeSH Terms] OR "acupuncture"[All Fields] OR "acupuncture therapy"[MeSH Terms] OR ("acupuncture"[All Fields] AND "therapy"[All Fields]) OR "acupuncture therapy"[All Fields] OR "electroacupuncture"[MeSH Terms] OR "electroacupuncture"[All Fields] OR "acupuncture, ear"[MeSH Terms] OR ("acupuncture"[All Fields] AND "ear"[All Fields]) OR "ear acupuncture"[All Fields] OR ("ear"[All Fields] AND "acupuncture"[All Fields]) OR ("auricular"[All Fields] AND "acupuncture"[All Fields]) OR "auricular acupuncture"[All Fields] OR "acupuncture points"[MeSH Terms] OR ("acupuncture"[All Fields] AND "points"[All Fields]) OR "acupuncture points"[All Fields])) AND (randomized controlled trial[pt] OR controlled clinical trial[pt] OR randomized[tiab] OR placebo[tiab] OR randomly[tiab] OR trial[tiab] OR groups[tiab] NOT (animals [mh] NOT humans [mh])) Filters: Publication date from 2013/01/01/ to 2017/12/31

Date Run: 29/01/18

1. **EMBASE**

('crossover procedure':de OR 'double-blind procedure':de OR 'randomized controlled trial':de OR 'single-blind procedure':de OR random*:de,ab,ti OR factorial*:de,ab,ti OR crossover*:de,ab,ti OR ((cross NEXT/1 over*):de,ab,ti) OR placebo*:de,ab,ti OR ((doubl* NEAR/1 blind*):de,ab,ti) OR ((singl* NEAR/1 blind*):de,ab,ti) OR assign*:de,ab,ti OR allocat*:de,ab,ti OR volunteer*:de,ab,ti) NOT (NOT human AND ('animals'/exp OR 'nonhuman' OR 'animal experiment')) AND ('acupuncture'/exp OR acupuncture OR 'auricular acupuncture' OR 'electroacupuncture'/exp OR 'electroacupuncture' OR 'acupuncture therapy'/exp OR 'acupuncture therapy' OR 'ear acupuncture'/exp OR 'ear acupuncture' OR (acupuncture AND point) OR acupoint) AND [embase]/lim AND (2013:py,af OR 2014:py,af OR 2015:py,af OR 2016:py,af OR 2017:py,af)

Date Run: 02/02/18

1. **Cochrane Controlled Register of Trials (CENTRAL)**

#1 MeSH descriptor: [Acupuncture Therapy] this term only (n=2835)

#2 MeSH descriptor: [Electroacupuncture] this term only (n=682)

#3 MeSH descriptor: [Acupuncture] this term only (n=169)

#4 MeSH descriptor: [Acupuncture, Ear] explode all trees (n=170)

#5 MeSH descriptor: [Acupuncture Points] explode all trees (n=1617)

#6 acupunct* or electroacupunct* or electro-acupunct* (Word variations have been searched) (n=12424)

#7 #1 or #2 or #3 or #4 or #5 or #6 (n=12424)

#8 #7 Publication Year from 2013 to 2017 (n=3421)

Date Run: 02/02/18
